# Ferroptosis and Liver Fibrosis

**DOI:** 10.7150/ijms.62903

**Published:** 2021-07-25

**Authors:** Qi Pan, Yi Luo, Qiang Xia, Kang He

**Affiliations:** Department of Liver Surgery, Renji Hospital, School of Medicine, Shanghai Jiao Tong University, Shanghai, China.

**Keywords:** ferroptosis, liver fibrosis, hepatic stellate cells

## Abstract

Ferroptosis is an iron-dependent form of regulated cell death, which is characterized by a large amount of lipid peroxide accumulation and the imbalance of redox state in cells. Ferroptosis is usually accompanied with the dysfunction of lipid repair enzyme (glutathione peroxidase 4, GPX4), large masses of iron accumulation and lipid peroxidation of polyunsaturated fatty acids (PUFAs). Ferroptosis is related to several signaling pathways, including amino acid and iron metabolism, ferritinophagy, cell adhesion and p53 and Keap1/Nrf2 signaling pathways. A number of studies have indicated that ferroptosis is closely associated with acute renal failure, tumor, ischemia and reperfusion injury, neurodegenerative diseases and liver fibrosis. Liver fibrosis, which has long been a global health problem, still lacks effective treatment till now, and the discovery of ferroptosis provides a new insight into addressing this issue.

## Overview of Ferroptosis

### Cell Death

Cell death is the irreversible endpoint of cells with notable morphological alterations. Loss of plasma membrane integrity, formation of apoptotic bodies or engulfment of cell debris by adjacent cells can be seen in several types of cell death 1. Typically the three canonical types of cell death are apoptosis, autophagic cell death and necrosis, but new modalities of non-canonical types have been identified, including paraptosis, pyroptosis, autoschizis and mitotic catastrophe 2.

Apoptosis is a form of programmed cell death (PCD) occurring in distinct pathophysiological contexts, which is characterized by cellular shrinkage, condensation and fragmentation of chromatin, pyknosis and plasma membrane blebbing. When apoptosis proceeds efficiently, membrane-enclosed structures containing mixtures of cell parts called apoptotic bodies are removed without leakage of intracellular contents, thus avoiding inflammation 3. Autophagic cell death is an evolutionarily conserved process that degrades cellular components in the lysosome, which is accompanied by large-scale autophagic vacuolization of the cytoplasm 4. Necrosis, however, is characterized by swelling of organelles and plasma membrane rupture, promoting inflammation to mediate pathogenesis in several diseases 5. Traditionally necrosis has been considered as an uncontrolled, accidental form of cell death, while evidence is accumulating that a form of regulated necrotic cell death, necroptosis, can be activated under certain conditions 6.

Ferroptosis is emerging as a new form of regulated necrosis, which is implicated in various hepatic diseases, including cancer, liver fibrosis and ischemia-reperfusion injury. Therefore, an in-depth understanding of the underlying mechanisms involved in the regulation of ferroptosis would allow improved targeting of ferroptosis and development of novel treatments for hepatic diseases.

### Iron Metabolism

As an essential trace element in the human body, iron is an important component of most cellular processes, such as DNA replication, mitochondrial respiration and cell signaling. Therefore, the disorder of iron metabolism, especially iron overload, plays an important role in the occurrence and development of several hepatic diseases 7. Iron can form both ferric (Fe^3+^) and ferrous (Fe^2+^) ions. After absorbed from duodenal villous epithelial cells from the diet, extracellular iron can be imported by transferrin and its carrier protein transferrin receptor (TFR) 8. TFR is sorted to endosomes after binding with transferrin, where the binding of transferrin and TFR as well as that of iron and transferrin are dissolved in acidic environment. Here again prior to release to cytosol via divalent metal transporter 1 (DMT1) Fe^3+^ in endosomes is reduced by ferrireductase six-transmembrane epithelial antigens of prostate 3 (STEAP3) 9. Fe^2+^ is then sorted to its destination for functional use or storage in labile iron pool (LIP) or in ferritin. Unnecessary Fe^2+^ may be excreted into extracellular circulation via ferroportin (FPN), the only iron exporter, while heme can be degraded by heme oxygenases (HOs) to recover Fe^2+^ 10. For example, HO-1 is a membrane-bound enzyme found in macrophages of the liver and spleen where its activity is essential for the recycling of heme-iron 11.

Iron metabolism is precisely regulated via iron regulatory proteins (IRPs, IRP1 and IRP2) and iron responsive element (IRE). IRP regulates iron metabolism mainly through post-transcriptional regulation of iron metabolism-related proteins, such as transporter receptor (TFRC) and ferritin 12.The binding of IRP to IRE can promote or inhibit the translation of mRNA of these genes, thus regulating the expression of protein 13. Iron homeostasis is also maintained by the hepcidin-FPN axis, which controls intestinal absorption of iron and internal iron recycling as well as systemic distribution 14. Silencing of TFRC gene or excessive expression of heat shock protein β1 (HSPB1) could significantly lower the intracellular iron content 15.

### Ferroptosis

Ferroptosis is an iron-dependent form of non-apoptotic cell death that is characterized by the accumulation of toxic lipid reactive oxygen species (ROS) 16. Its name was coined in 2012 as the discovery of erastin and Ras selective lethal small molecules (RSLs) selectively induced a non-apoptotic programmed cell death in cancer cells which can be blocked by iron chelators. Ferroptosis cells exhibit changed mitochondrial morphology and cristae structure. Smaller than normal mitochondria with condensed membrane density, reduction or vanishing of mitochondrial crista and rupture of the outer mitochondrial membrane has been observed in ferroptosis following erastin treatment in cancer cells. In contrast, no chromatin margination or nuclear condensation is observed and the structural integrity of the nucleus is retained in ferroptotic cells 17.

Although iron is an inorganic essential nutrient for cell proliferation, excessive iron generates catalytic Fe^2+^ in the body, which causes the Fenton reaction (Fe^2+^+ H2O2→Fe^3+^ + •OH + OH^-^). The generated hydroxyl radicals (•OH) directly attack lipids, which leads to the peroxidation of PUFAs and contributes to ferroptotic cell death 18. There is no doubt about the key role of iron metabolism in the process of ferroptosis although the exact mechanism remains obscure, because iron chelators block ferroptosis *in vivo* and *in vitro* 19. Increased LIP is usually observed during the induction of ferroptosis and excessive iron can directly induce ferroptosis 20. Exogenous supplementation of iron increases the sensitivity of cancer cells to ferroptosis inducers, such as erastin 21.

### Molecular Mechanisms of Ferroptosis

GPX4 is a monomeric glutathione peroxidase with a unique ability to reduce hydroperoxide in complex lipids and plays a key role in enzymatic defense against membrane lipid peroxidation. Deficiency of GPX4 is found to induce ferroptotic cell death 22. Glutathione (GSH), the reducing agent required for GPX4 function, plays a crucial role in ferroptosis. GSH reduces reactive nitrogen and ROS under the action of GPX4, and ferroptosis can be induced by the inactivation of GPX4. Loss of GPX4 activity is mediated by direct or indirect mechanism such as the depletion of GSH.

Cystine/glutamate antiporter system (system X_c_-) is a cystine/glutamate exchange transporter acting in a Na^+^-dependent way, which is a disulfide-linked heterodimer comprising a light-chain subunit (xCT, SLC7A11) and a heavy-chain subunit (CD98hc, SLC3A2) 23. In system X_c_-, sodium-independent antiporter imports cystine and exports glutamate in an ATP-dependent manner. Cystine is rapidly reduced to cysteine once internalized, which is involved in the synthesis of GSH 24. Inhibiting the activity of system X_c_- will affect the synthesis of GSH by inhibiting the absorption of cystine, which leads to a decrease in GPX activity and results in a reduction in cell antioxidant capacity, accumulation of lipid ROS, and ferroptosis 25. An in-depth study of RSLs showed that GPX4 was a target protein of RSL3. After binding to GPX4, RSL3 inactivates GPX4 to induce ROS production from lipid peroxidation and causes ferroptosis, which can be reversed by RSL3 inhibitors 26. A series of small molecule inducers, namely ferroptosis-inducing agents (FINs) were discovered in a screening to find ferroptosis-inducing compounds. Like RSL3, FIN compounds including DPI family members (DPI7/10/12/13/17/18/19) and FIN56 directly inhibit GPX4 activity without GSH depletion 27. Inactivation of GPX4 by GSH depletion is found to trigger ferroptosis by accumulation of ROS from lipid peroxidation 28.

### Regulators of Ferroptosis

The execution of ferroptosis is closely related to ROS, iron and PUFAs, therefore many genes and signaling pathways related to ROS production, iron metabolism and lipid synthesis have been found to probably regulate the vulnerability to ferroptosis 29. The main cellular mop for ROS is GSH, and lack of GSH blunts the activation of GPX4, which plays a regulatory role in ferroptotic cell death 30. Genes involved in iron metabolism and lipid peroxidation regulate the occurrence and development of ferroptosis. For example, activation of autophagy supplies available labile iron via nuclear receptor coactivator 4 (NCOA4) mediated ferritinophagy to the process of ferroptosis. NCOA4 deletion inhibits ferroptosis and NCOA4 over-expression increases sensitivity to ferroptosis. Thus, NCOA4-mediated ferritin degradation is involved in ferroptosis 31. IRP-IRE axis regulates the thanslation of mRNAs that affect iron homeostasis as described above. The HO-1 inducer hemin accelerates erastin-induced ferroptotic cell death while the HO-1 inhibitor zinc protoporphyrin IX (ZnPP) prevents ferroptosis. Ferroptosis suppressor protein 1 (FSP1) is recruited to the plasma membrane where it functions as an oxidoreductase that reduces coenzyme Q10 (CoQ) and generate a lipophilic radical-trapping oxidant which halts the propagation of lipid ROS and development of ferroptosis. These findings define a new ferroptosis suppression pathway which acts in parallel to the canonical GPX4 pathway 32. Free PUFAs, which are substrates of lipoxygenases (LOXs), are susceptible to lipid peroxidation and are necessary for the execution of ferroptosis. Genetic depletion of LOXs can protect against ferroptosis, suggesting LOXs contribute to ferroptosis and several ferroptosis inhibitors can inhibit LOXs activity in some contexts 33. It should be noted that ACSL4(acyl-CoA synthetase long-chain family member 4) is also an essential component of fatty acid metabolism, which is responsible for the esterification of CoA to free PUFAs, thus playing a role in the execution of ferroptosis 34. It is demonstrated that ACSL4 can modulate lipogenesis by accumulating intracellular lipid and is involved in the process of inflammation, thus contributing to the development of liver fibrosis^35^.

A variety of different substances can lead to the induction of ferroptosis, such as erastin, sorafenib and RSL3. Most of ferroptosis inducers were discovered before the biological process was identified, which have been classified into four types according to the mechanism underlying ferroptosis induction. Class 1 ferroptosis inducers, such as erastin, sulfasalazine and sorafenib, inhibit system X_c_- and deplete cellular cysteine. Cysteine depletion in cells results in the inhibition of the biosynthesis of GSH and the loss of GPX4 activity, leading to the accumulation of lipid ROS and eventual ferroptosis 36. Class 2 ferroptosis inducers, such as RSL3, RSL5 and DPI compounds, inhibit GPX4 directly. These substances inhibit GPX4 activity by covalently interacting with the active site of GPX4. Eventually, ferroptotic cell death occurs due to the accumulation of lipid ROS 37. Class 3 ferroptosis inducers, such as FIN56, CIL56 and statins, promote the degradation of GPX4 protein and deplete endogenous antioxidant coenzyme CoQ via the mevalonate pathway. CoQ is a well-known component in the mitochondrial electron transport chain and is essential for preventing the accumulation of lipid ROS. In addition, statins enhance the sensitivity of the cell to ferroptosis by inhibiting HMG-CoA reductase, which is involved in the mevalonate pathway 38. Class 4 ferroptosis inducers, such as FINO_2_ and 1,2-dioxolane, cause direct oxidation of LIP and inactivation of GPX4, consequentially causing extensive lipid peroxidation 39.

With the increasing relevance of ferroptosis as a pharmacological target, various ferroptosis inhibitors, such as iron chelators and ferrostatin-1, have been identified. These compounds can be classified into two types since lipid peroxidation can occur via enzymatic and non-enzymatic pathways. Class 1 ferroptosis inhibitors, such as deferoxamine mesylate, suppress iron accumulation and inactivate LOX by removal of the active site iron 40. Class 2 ferroptosis inhibitors, such as liproxstatin-1, ferrostatin-1 and vitamin E, prevent lipid peroxidation 41. In addition, there are other substances that have not been classified. 2-mercaptoethanol bypasses system X_c_- and inhibits ferroptosis by reducing cystine to cysteine, and cycloheximide suppresses ferroptotic cell death induced by system X_c_- inhibition 42, 43.

## Ferroptosis and Liver Fibrosis

### Overview of Liver Fibrosis

Liver fibrosis is characterized by excessive accumulation of extracellular matrix (ECM) proteins, primarily fibrillar collagen-I, in response to persistent liver injury. Liver fibrosis can be caused by alcohol abuse, non-alcoholic fatty liver disease and chronic infection of hepatitis B virus (HBV) or hepatitis C virus (HCV). Cirrhosis is the terminal stage of liver fibrosis and lethal complications of cirrhosis include liver failure, hepatocellular carcinoma (HCC) and hepatic encephalopathy 44. Activation of hepatic stellate cells (HSCs) is the major driver of liver fibrogenesis. Transdifferentiation of HSCs is the major cellular source of myofibroblasts which secretes ECM proteins. Quiescent HSCs transdifferentiate into proliferative and migratory myofibroblasts, which are plausibly linked to liver fibrosis 45. It is indicated that chronic liver injury can be terminated, and liver fibrosis and even cirrhosis can be reversed by therapeutic intervention aiming at the primary disease etiology. Regression of liver fibrosis is accompanied with reduced or loss of activated HSCs, which suggests that deactivating or reducing HSCs could be an antifibrotic strategy irrespective of the cause of liver injury 46. Enhancing autophagy may help prevent the progression of a number of liver diseases, while targeting specific liver cells must be considered in some situations such as liver fibrosis, as autophagy may display opposing functions depending on different cell types 47. The only effective clinical treatment is liver transplantation for patients who have progressed to cirrhosis. Therefore, it is urgent to find new therapeutic strategies and agents for anti-fibrotic therapy, and inhibition of HSCs activation is an effective strategy to treat and prevent liver fibrosis 48.

### Ferroptosis and Liver Fibrosis

Iron is abundant in HSCs, which is a prerequisite for ferroptotic cell death 33. It appears that ferroptosis functions as a two-edged sword in the occurrence and development of liver fibrosis. It is found that excessive hepatic iron deposition and ferroptosis potentiates liver fibrosis induced by acetaminophen in mice populations, which can be regressed by ferrostatin-1 4. Therefore, ferroptosis could promote the formation of liver fibrosis 10.

However, it is reported that ferroptosis can be considered as a new strategy to eliminate HSCs and ameliorate liver fibrosis. In mice, treatment with erastin and sorafenib alleviated murine liver fibrosis by inducing ferroptosis, which regulates autophagy signaling pathway in HSCs. RNA-binding protein, such as ZFP36/TTP (ZFP36 ring finger protein) and ELAVL1/HuR, plays a crucial role in regulating ferroptosis in HSCs. ZFP36 helps protect against ferroptosis, whereas ELAVL1 plasmid contributes to ferroptotic cell death 49. Magnesium isoglycyrrhizinate (MgIG) treatment markedly reduced fibrotic scar formation and attenuated liver injury in the rat model of hepatic fibrosis. HO-1 was found in the upstream position of MgIG-induced HSCs ferroptosis, and HO-1 knockdown evidently blocked ferroptotic cell death in HSCs and in turn exacerbated liver fibrosis 50. Artemether and artesunate, derivatives of artemisinin, are first-line treatment for malaria, which exert effects of anti-inflammation and immune regulation. P53 pathway plays a vital role in cellular ferroptosis in response to various diseases and documents suggested that P53 was critical for inducing ferroptosis 38. Artemether could dose- and time-dependently promote the expression and nuclear import of P53. Western blot analysis showed that siRNA-mediated P53 knockdown abrogated artemether-induced antifibrosis effects. By decreasing GPX4 and SLC7A11 expression, artemether remarkably increased the levels of iron and ROS and promoted ferroptosis in activated HSCs 51. Evidences have shown that artesunate could induce the accumulation of ferroptotic activated HSCs in carbon tetrachloride (CCl_4_)-induced mouse fibrotic liver and alleviate liver fibrosis by regulating ferritinophagy-mediated ferroptosis in HSCs 52. Hepatitis B virus X protein (HBx) is critical for liver fibrosis development, which alleviates cell death of HSCs by inhibiting ferroptosis. Chrysophanol helps reverse HBx-mediated inhibition of ferroptosisis via downregulation of GPX4 and SLC7A11. Therefore, chrysophanol could attenuate HBx-induced HSCs activation and ameliorate liver fibrosis 53. Recently, dihydroorotate dehydrogenase (DHODH) was found to operate in parallel to GPX4 in mitochondrials to inhibit ferroptosis, which reduces ubiquinone to ubiquinol. Ubiquinol then acts as a radical-trapping antioxidant to eliminate lipid ROS. As a result, inactivation of DHODH may induce or ferroptosis in hepatic cells. Brequinar, the DHODH inhibitor, shows promise for suppression or even treatment of liver fibrosis 54. Nowadays the idea of ferroptosis-inducing therapy is becoming consistent in the field of liver fibrosis treatment. However, the relationship between ferroptosis and liver fibrogenesis is still unknown, therefore it is of great theoretical significance and practical value to explore the mechanism of ferroptosis and its role in liver fibrogenesis.

## Conclusion

**Figure 1:**
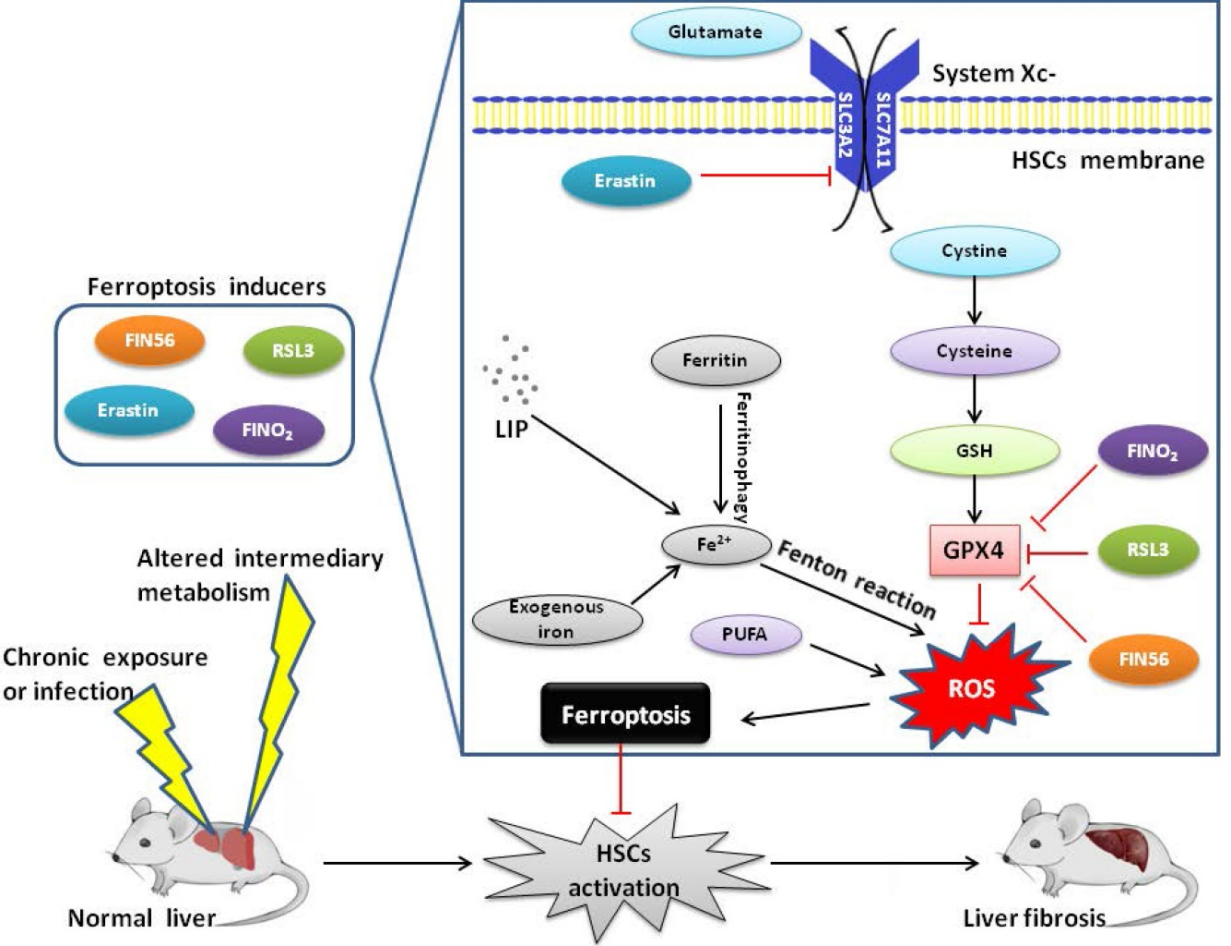
Mechanism of ferroptosis and liver fibrosis

In summary, ferroptosis is a new type of programmed cell death, which plays an important regulatory role in the occurrence and development of liver diseases (Figure 1). Currently, pharmacotherapy remains the mainstream approach for patients with liver fibrosis. However, the discovery of ferroptosis has opened up a new platform in the field of liver fibrosis. Although research on ferroptosis is still in its infancy, several molecules have recently been identified to regulate ferroptosis. Thus, the most significant objective is to identify new molecular markers for ferroptosis and downstream signaling pathways which distinguish ferroptosis from other types of PCD. However, the effect of ferroptosis on liver fibrosis is not clear. Therefore, further research is needed to determine the effect of ferroptosis on liver pathophysiology and more efforts are required to explore the underlying regulatory mechanisms of ferroptosis in more disease-specific contexts. Additionally, an improved understanding of the role of ferroptosis in liver fibrosis will create a new opportunity for diagnosis and propose effective and highly targeted therapies, which is also the future direction of ferroptosis research.
